# Evaluation of parylene derivatives for use as biomaterials for human astrocyte cell patterning

**DOI:** 10.1371/journal.pone.0218850

**Published:** 2019-06-25

**Authors:** Brad J. Raos, M. Cather Simpson, Colin S. Doyle, E. Scott Graham, Charles P. Unsworth

**Affiliations:** 1 Department of Engineering Science, The University of Auckland, Auckland, New Zealand; 2 Departments of Chemistry & Physics, The University of Auckland, Auckland, New Zealand; 3 The MacDiarmid Institute for Advanced Materials and Nanotechnology, Wellington, New Zealand; 4 The Dodd Walls Centre for Photonic and Quantum Technologies, Dunedin, New Zealand; 5 Department of Chemical and Materials Engineering, The University of Auckland, Auckland, New Zealand; 6 Department of Molecular Medicine and Pathology, and Centre for Brain Research, The University of Auckland, Auckland, New Zealand; Massachusetts Institute of Technology, UNITED STATES

## Abstract

Cell patterning is becoming increasingly popular in neuroscience because it allows for the control in the location and connectivity of cells. A recently developed cell patterning technology uses patterns of an organic polymer, parylene-C, on a background of SiO_2_. When cells are cultured on the parylene-C/SiO_2_ substrate they conform to the underlying parylene-C geometry. Parylene-C is, however, just one member of a family of parylene polymers that have varying chemical and physical properties. In this work, we investigate whether two commercially available mainstream parylene derivatives, parylene-D, parylene-N and a more recent parylene derivative, parylene-HT to determine if they enable higher fidelity hNT astrocyte cell patterning compared to parylene-C. We demonstrate that all parylene derivatives are compatible with the existing laser fabrication method. We then demonstrate that parylene-HT, parylene-D and parylene-N are suitable for use as an hNT astrocyte cell attractive substrate and result in an equal quality of patterning compared to parylene-C. This work supports the use of alternative parylene derivatives for applications where their different physical and chemical properties are more suitable.

## Introduction

Patterning of *in vitro* cell culture surfaces facilitates spatial control over the growth of cells. In contrast to typical cell culture, patterned cultures offer the opportunity to better represent more complex patterns of connectivity that reflect the *in vivo* environment [1, 2]. Cell patterning can be achieved by tuning the physical and chemical properties of polymers so that they form cell repulsive and cell attractive surface [3–5]. A cell patterning technique that was recently developed by Delivopoulos et al. demonstrated how geometric patterns of the polymer parylene-C on a background of non-permissive SiO_2_ could be exploited to generate patterned cell cultures [6–9].

The parylene-C/SiO_2_ platform has found utility in patterning neurons and glia, motivated by the need to precisely control the location of neuronal cell bodies at the interface of multi-electrode arrays and to control the connectivity within networks of neurons and astrocytes [6–12]. Delivopoulos et al. and Unsworth et al. generated patterned co-cultures of murine neurons and glia [6, 8]. Similarly, Hughes et al. demonstrated patterning of LUHMES neuronal networks by first patterning a template of glial-like stem cells on parylene-C/SiO_2_ substrates [10]. More recently, human neurons and astrocytes, which were derived from the NTera2.D1 (hNT/NT2) cell line, have been patterned by Unsworth et al., Jordan et al., and Raos et al. [7, 9, 11, 12]. The NT2 (hNT) cell line provides a readily available source of neurons and astrocytes that are more applicable to human physiology than primary cells of non-human origin [13]. hNT neurons and astrocytes have been extensively characterised and validated as a model for primary human neurons and astrocytes [14–21]. Unsworth et al. used the parylene-C/SiO_2_ platform to isolate single hNT astrocytes *in vitro*. That work was expanded on by Raos et al. who demonstrated that the parylene-C/SiO_2_ platform can be used to selectively control the patterning of hNT astrocytes *in vitro*. Raos et al. demonstrated that patterned clusters of hNT astrocytes exhibited spatio-temporally clustered Ca^2+^ transients that were not observed in non-patterned cultures [11]. Similarly, Jordan et al. used parylene-C that was deposited into grids of photolithographically defined SiO_2_ trenches to demonstrate that functionality of the hNT astrocytes through ATP evoked calcium signalling was dependent on the grid spacing [12]. Raos et al. recently presented a modification to the parylene-C/SiO_2_ cell patterning platform where polyethylene glycol was selectively attached to the SiO_2_ regions of parylene-C/SiO_2_ substrates [22] mitigating the need for serum. Furthermore, Raos et al. developed a method to etch the desired pattern into the substrate using IR laser ablative micromachining [23] for rapid prototyping, avoiding the expensive photolithographic step.

**Fig 1:**
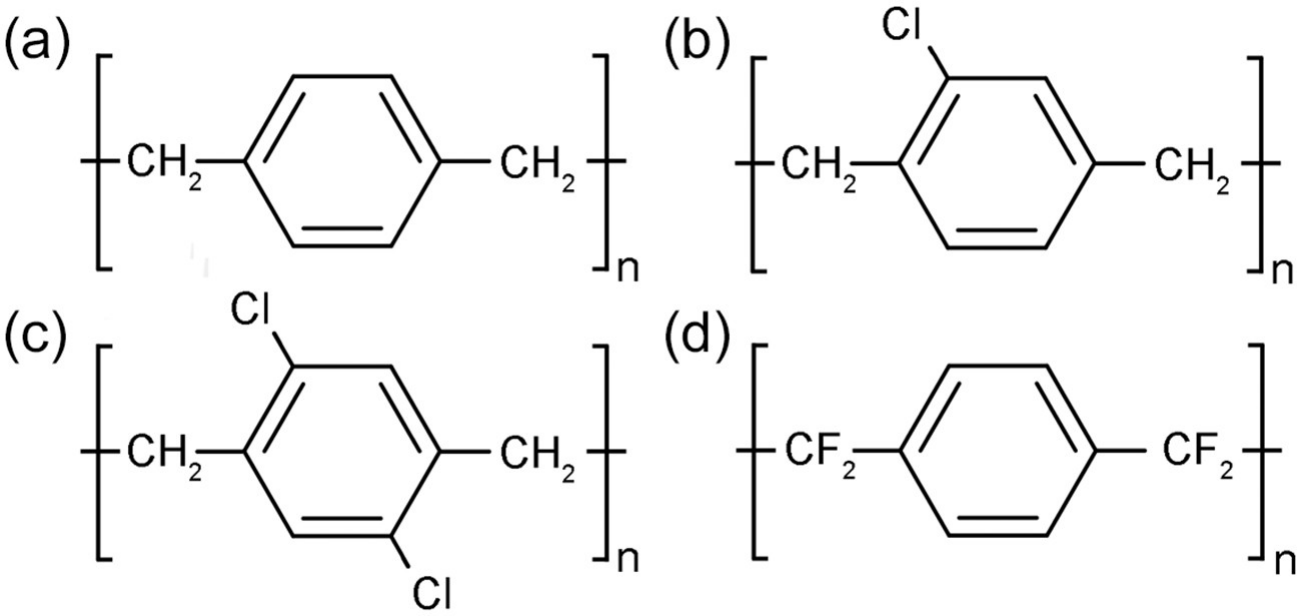
Chemical structure of the parylene derivatives. (a) Parylene-N, (b) Parylene-C, (c) Parylene-D and (d) Parylene-HT.

Contemporary parylene/SiO_2_ literature has exclusively used parylene-C as the cell attractive material. Parylene-C, however, is part of a larger family of organic polymers that differ chemically in the substitutions of the underivatised parylene structure. The chemical structure of underivatised parylene, referred to commercially as parylene-N, is shown in Fig 1(a). Similarly, the halogenated derivatives parylene-C, parylene-D and, the more recently developed parylene-HT [24], are shown in Fig 1(b), 1(c) and 1(d), respectively.

Parylene derivatives differ in their chemical and physical properties [25]. Parylene-HT exhibits a lower auto-fluorescence [26], while both parylene-HT and parylene-N have superior crevice penetration ability, increasing their utility as an internal protective coatings on bio-MEMS devices [24]. Parylene-HT has greater UV and thermal stability due to the lack of a labile chlorine substitution and the greater enthalpy of the carbon-fluorine bond [24].

While a complete mechanism that describes cell patterning on parylene/SiO_2_ surfaces has not been fully developed, it is hypothesized to involve differences in the hydrophilic/hydrophobic interactions between serum proteins and the respective surfaces. Those differences result in differential protein adsorption. Although the exact combination of proteins has not been elucidated, bovine serum albumin, fibronectin and vitronectin are thought to adsorb to both parylene-C and SiO_2_ surfaces in different amounts and conformations [6, 27, 28]. Subsequent interactions between cell specific integrin receptors and the available binding domains on the adsorbed protein render the SiO_2_ surface non-permissive while leaving the parylene-C cytophilic [27].

The effect of the hydrophilic/hydrophobic nature of parylene-C on cell patterning has been investigated indirectly. Delivopoulos et al. reported that the exposure of parylene-C/SiO_2_ substrates to UV radiation inhibited cell patterning. The loss of cell patterning was hypothesized to be the result of UV induced oxidation of the parylene-C surface [29]. A similar effect was observed by Raos et al. who investigated cell patterning on parylene-C/SiO_2_ substrates that were fabricated using UV or IR laser ablative micromachining [23]. Substrates that were fabricated using a UV laser did not result in cell patterning. In contrast, those that were fabricated with an IR laser did result in cell patterning. Raos et al. demonstrated that the UV laser resulted in the incorporation of oxygen to the parylene-C surface. That surface oxidation was hypothesized to account for the failed cell patterning. Although Delivopoulos et al. and Raos et al. did not measure the effect on surface hydrophilicity, oxidation of parylene is known to introduce carbonyl, carboxyl and hydroxyl groups to the surface [30]. Oxygen containing functional groups increase the surface energy, thereby making the surface more hydrophilic [31–33].

The procedure for preparing parylene-C/SiO_2_ substrates for cell patterning involves immersion in piranha acid, a strong oxidizing agent. Hughes et al. has demonstrated that the piranha acid immersion is necessary to enable cell patterning [27]. In the absence of piranha acid treatment HEK293 cells showed no preference for parylene-C surfaces over SiO_2_ surfaces. Hughes et al. has also, however, demonstrated that the piranha acid treatment increases the hydrophilic nature of parylene-C [34].

The work of Delivopoulos et al. and Raos et al. suggests that significant increases in the hydrophilicity of parylene-C are detrimental to cell patterning, however, Hughes et al. suggests that a small increase in hydrophilicity is inevitable as a result of the piranha acid treatment. It is well known that moderately hydrophilic surfaces are optimal for cell adhesion. As surfaces become highly hydrophilic or hydrophobic, cell attachment decreases [33, 35, 36]. Untreated parylene-HT, parylene-D, parylene-N and parylene-C have reported water contact angles of ~100, 97, 79, and 87°, respectively [37, 38]. Therefore, we sought to investigate whether using an alternative parylene derivative that is either initially more or less hydrophilic than parylene-C would improve the fidelity of cell patterning on the parylene/SiO_2_ platform.

Parylene-C has been evaluated extensively as a cell culture substrate for a wide variety of cell types, including human and murine fibroblasts [39, 40], embryonic stem cells [41], the PC12 [42] and HEK293 [27] cell lines and primary murine neurons and astrocytes [6]. In contrast, parylene-HT, parylene-D and parylene-N have been less extensively evaluated. Parylene-N has recently been used for the culture of osteoblast like cells [43], while parylene-HT has been used as a substrate for 3T3 murine fibroblast cells [44].

This article is motivated by the reported differences in the hydrophilicity of parylene-HT, parylene-D, parylene-N and the fact that the cell patterning mechanism relies on specific hydrophilic/hydrophobic surface interactions with serum proteins. In this paper, we investigate whether alternative parylene derivatives are compatible with existing fabrication techniques and whether they result in improved cell patterning.

## Methods

### Design and fabrication of parylene/SiO_2_ substrates

Silicon wafers were oxidized in a furnace at 950 °C for 40 min, under a flow of 1.88 and 1.25 sccm of H_2_ and O_2_ respectively, to produce a 200 nm SiO_2_ layer. The depth of the SiO_2_ layer was confirmed with a Nanometrics NanoSpec/AFT Microarea gauge. Parylene deposition was performed by Speciality Coating Systems which deposited a 100 nm layer of either parylene-HT, parylene-D, parylene-N or parylene-C onto separate wafers.

**Fig 2:**
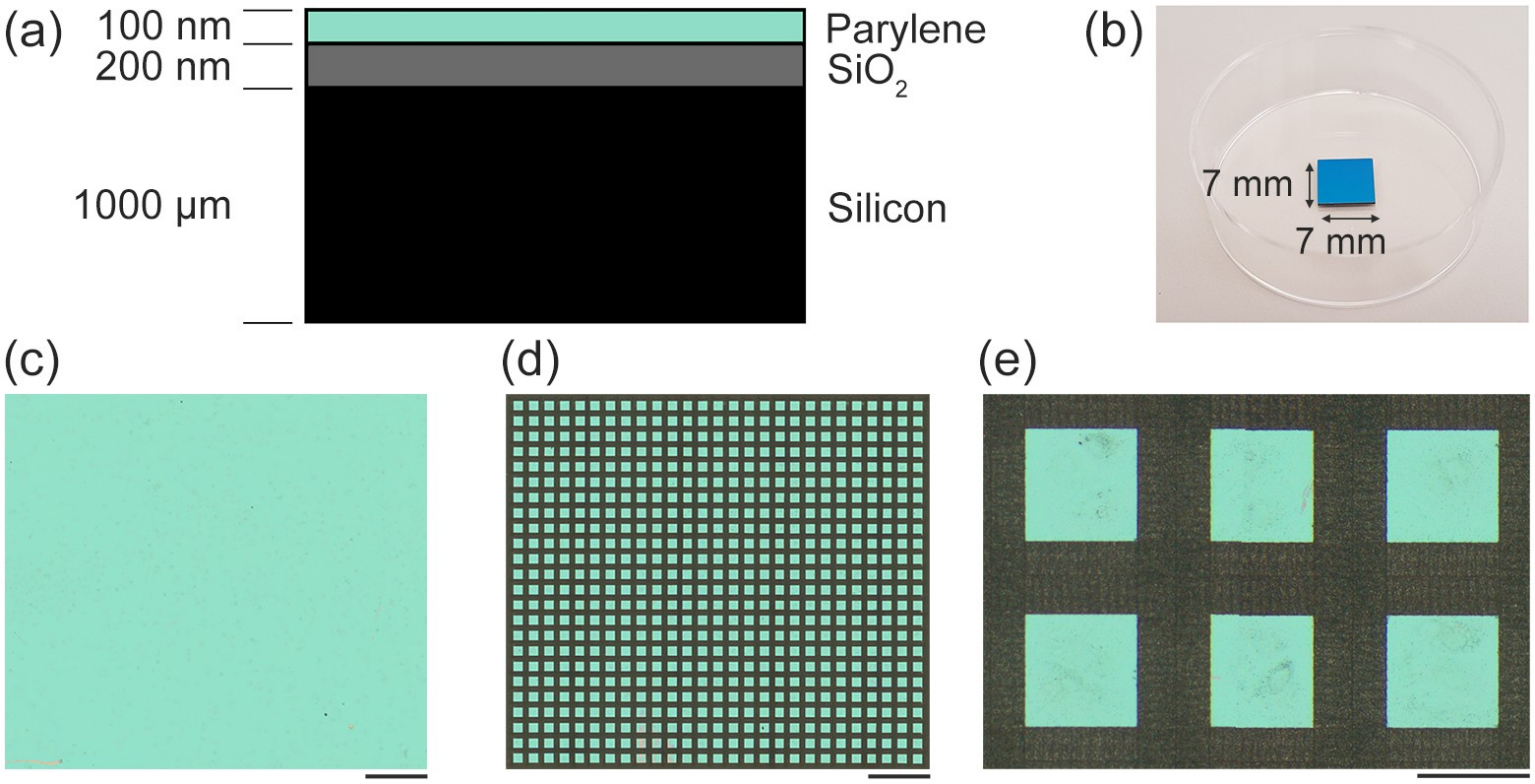
Description of parylene/SiO_2_ substrates. (a) Vertical cross-section of a parylene/SiO_2_ substrate (not to scale). (b) Image of a single blank parylene/SiO_2_ sample. (c) An example of a parylene/SiO_2_ substrate before and (d and e) after femtosecond IR laser micromachining. Green areas consist of parylene, whereas the grey areas consist of SiO_2_. Scale-bar = 1 mm in (c, d) and Scale-bar = 150 μm in (e).

Fig 2, shows an example of the parylene/SiO_2_ substrates we used in this work. The blank parylene/SiO_2_ substrates, shown in Fig 2(b) and 2(c), were modified by femtosecond laser ablative micromachining using a method that has been previously described by Raos et al. [23]. Briefly, a Coherent Legend femtosecond-pulsed laser (800 nm, 100 fs pulses, repetition rate 500 Hz) was directed through a square, chrome-plated quartz mask towards a computer-controlled motorized stage and focussed at a spot size of 50 μm. As the sample was moved relative to the laser beam the desired pattern was ablated from the surface, as is shown in Fig 2(d) and 2(e). The cell-patterning substrate consisted of a grid of equally spaced square nodes of parylene surrounded by SiO_2_ (Fig 2(d) and 2(e)). In this work we used square 150 x 150 μm parylene nodes with an inter-node distance of 95 μm. After fabrication, the substrates were kept in light-proof boxes until they were used because exposure to UV light is known to inhibit cell patterning on the parylene-C/SiO_2_ platform.

The patterned parylene-C/SiO_2_ samples were cleaned with a 30-second acetone wash and rinsed in Milli-Q water. The samples were then cleaned with piranha acid, consisting of a 5:3 ratio of hydrogen peroxide (30%) and sulphuric acid (98%), for 10 min and subsequently rinsed three times in Milli-Q water. Immediately after the piranha acid treatment, the samples were sterilized by immersion in 0.5 mL antibiotic/antimycotic solution (1% Penicillin-Streptomycin) and incubated for 1 h at 37 °C. The samples were then rinsed in PBS, immersed in 100% foetal bovine serum (FBS) and incubated for 3 hours in order to activate the patterns.

### Assessment of laser fabrication quality with XPS

XPS spectra were recorded on a Kratos Axis Ultra DLD spectrometer (Kratos Analytical, Manchester UK). Survey spectra and Carbon 1s (C 1s) elemental spectra were collected from 300 x 700 μm areas on the parylene and SiO_2_ regions of each sample. Survey spectra were collected with a pass energy of 160 eV and dwell time of 138 ms from five 180 s sweeps. Elemental spectra were collected with a pass energy of 20 eV and a dwell time of 260 ms from fifteen 60 seconds sweeps. The vacuum chamber pressure was kept below 2 x 10^−9^ Torr. Samples were illuminated with monochromated Aluminium *K*_*α*_ X-rays at 1486.69 eV and analysed with charge neutralisation.

### Astrocyte differentiation and seeding on patterned substrates

The NTera2.D1 (hNT/NT2) cell line was obtained from American Type Culture Collection (CRL-1973). NT2 cells were differentiated into astrocytes using a protocol that has been described extensively [7, 14, 16, 45–47]. Briefly, cells were cultured in DMEM:F12 that was supplemented with 1% Penicillin-Streptomycin-Glutamine, 5% or 10% FBS and specific combinations of retinoic acid (RA), uridine (Urd), 5-fluoro-2’-deoxyuridine (FUdR) and *β*-D-arabinofuranoside (AraC). All media changes were performed every 2–3 days. Cell cultures were maintained at 37°C, 5% CO_2_. Initially, NT2 cells were cultured in media that contained 10% FBS, were plated in Petri dishes at 6 x 10^6^ cells per dish and were treated with RA (10 μM) for 2 weeks. The cells were then transferred to T75 flasks and treated with RA for a further 7–10 days. Cells with neuronal morphology were removed by partial trypsinization and the remaining non-neuronal cells were replated into T75 flasks. The remaining cells were subsequently cultured in 5% FBS media and treated with Urd (10 μM), FUdR (10 μM) and AraC (1 μM) for 12 days, followed by Urd and FUdR (10 μM) for 13 days, followed by Urd (10 μM) for 8 days. Any cells with a neuronal morphology were again removed by selective trypsinization and discarded. The astrocytes were harvested by trypsinization for seeding onto patterned parylene/SiO_2_ substrates.

Following serum incubation and prior to seeding the parylene/SiO_2_, samples were gently rinsed in PBS. hNT astrocytes were then plated onto the samples at 50 cells per mm^2^.

### Fluorescence imaging and labelling

The quality of cell patterning on parylene/SiO_2_ substrates was evaluated 48 hours after seeding. Astrocytes were fluorescently labelled to visualise the cytoplasm (CMFDA, 1 μM, for 30 min), and the nuclei (Hoechst 33258, 1.6 μg mL^-1^, for 10 min). The cells were fixed with 3.6% PFA for 10 min at ambient conditions and washed 3x in PBS. Samples were imaged on an Olympus BX53 that was equipped with a motorized stage, using GFP and DAPI (470-495/550 nm and 360-370/410, Em/Ex) fluorescence filters. Greyscale 8-bit images were captured on Olympus XC50 camera at 10x magnification in a grid mosaic pattern covering the entire sample. Custom software written in Matlab (2014b, The MathWorks Inc., Natick, MA) was used for further processing.

Visualisation of the astrocyte Ca^2+^ signalling was evaluated using time-lapse microscopy and a fluorescent Ca^2+^ probe using a method we developed previously (see Raos et al. for a detailed description of the methods used [11]). Briefly, astrocytes were labelled with Fluo-4 (1 μm in 1% FBS/Fluorobrite DMEM) for 30 min at 37°C and 5% CO_2_. The parylene/SiO_2_ substrates were then transferred to fresh 1% FBS/Fluorobrite DMEM and imaged on the microscope setup that was previously described with the addition of an environmental chamber to maintain the atmosphere at 37°C and 5% CO_2_. Fluo-4 was imaged using the GFP fluorescence filter. Images were captured with 500 ms exposures at 0.5 Hz for 400 s.

### Image processing

Images of the astrocytes on the parylene/SiO_2_ substrates were processed into binary masks that represented the astrocyte cytoplasm, the astrocyte nuclei, and the parylene nodes (see Raos et al. and Unsworth et al. [8, 11] for a complete description of the image processing algorithm). Briefly, the fluorescence images were pre-processed by applying a histogram equalization routine and a 5x5 Wiener filter. The images were converted to binary masks using a manually set threshold. The binary masks were then morphologically opened using a 7-pixel disk structural element. The masks were then used to quantify the level of cell patterning using four metrics that have previously been applied to the parylene/SiO_2_ platform:

1. The parylene adhesion index (PAI), represents the fraction of parylene area that is covered by cellular content [6, 27]. Cultures with a high PAI reflect parylene surfaces that are favourable to cell growth.
2. The silicon repulsion index (SRI), represents the fraction of the SiO_2_ area that is not covered by cellular content [6, 27]. Cultures with a high SRI reflect high fidelity patterning.
3. The node isolation index (NIX), represents how well astrocyte clusters on an individual node are isolated from one another. The NIX for an individual node is calculated as the inverse of the number of nodes that are connected by cellular content [11]. The optimal NIX values is 1, representing a patterned culture where all nodes are isolated from one another. The NIX value increases as the patterning fidelity decreases because cells grow in the SiO_2_ and physically connect clusters of cells on adjacent nodes.
4. The mean number of astrocytes in an isolated cluster of cells.

Statistical analysis of the patterned cultures was performed in MATLAB. Null hypotheses and p-values are reported in the text where relevant. The Bonferroni correction was applied where multiple comparisons were performed.

Images from the Ca^2+^ visualisation experiments were imported into MATLAB, regions of interest (ROI) for each astrocyte were obtained by manually tracing the cell boundaries, and the mean fluorescence was measured for each ROI at every time point. Background subtraction was performed on each fluorescence trace using the method of Jia et al. [48].

## Results and discussion

In this section, we evaluate whether parylene-HT, parylene-D and parylene-N are compatible with existing protocols for fabricating parylene/SiO_2_ substrates, and compare the quality of cell patterning achieved with parylene-C substrates.

### Assessment of laser fabrication quality

**Fig 3:**
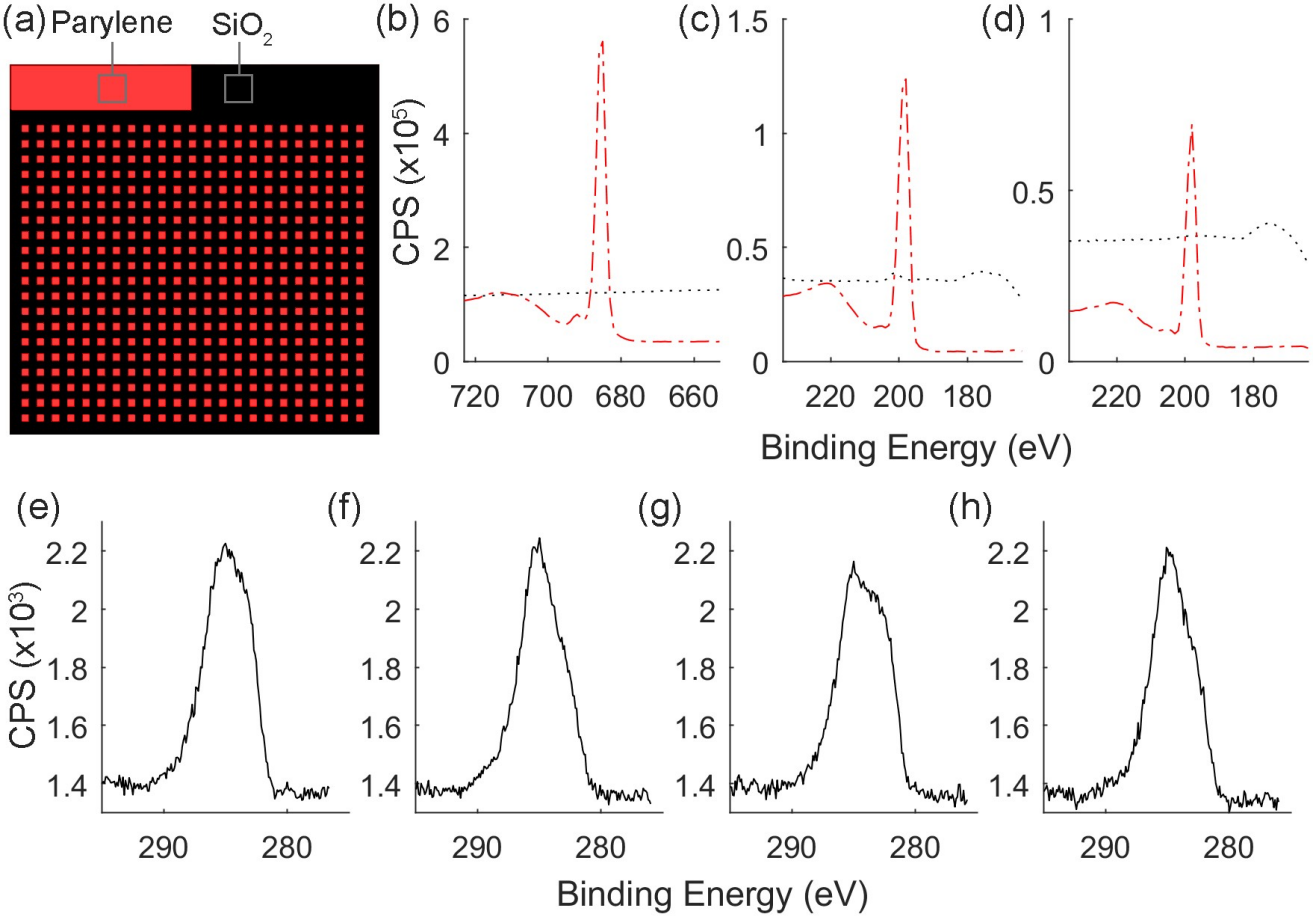
XPS analysis of parylene/SiO_2_ substrates that were manufactured by femtosecond laser ablation. (a) Schematic of the parylene/SiO_2_ substrates indicating the location of spectra collection. (b) Fluorine 1s spectra of parylene-HT (Red, dashed line), and ablated SiO_2_ (Black, dotted line), (c) Chlorine 2p spectra of parylene-D (Red, dashed line), and ablated SiO_2_ (Black, dotted line), (d) Chlorine 2p spectra of parylene-C (Red, dashed line), and ablated SiO_2_ (Black, dotted line). (e, f, g, h) Carbon 1s spectra collected from ablated SiO_2_ regions of (e) parylene-HT, (f) parylene-D, (g) parylene-C and (h) parylene-N substrates.

XPS spectra were collected from both parylene and ablated SiO_2_ areas of the substrates in order to verify that each parylene derivative had been completely removed. Fig 3 demonstrates the successful removal of parylene-HT, parylene-D and parylene-C by the absence of fluorine and chlorine that are present in the respective polymers.

Parylene-HT, parylene-D and parylene-C contain characteristic halogen elements that would not be present on ablated SiO_2_ regions unless the parylene had not been completely removed. Fig 3(b) demonstrates complete ablation of parylene-HT by the absence of the fluorine 1s peak at 688 eV, while Fig 3(c) and 3(d) demonstrate the complete ablation of parylene-D and parylene-C, respectively, by the absence of the chlorine 2p peak at 199 eV. Parylene-N, however, does not contain any characteristic elements to verify its complete removal. Verification of the complete removal of Parylene-N was performed by comparing the amount and chemical nature of the carbon present on the ablated SiO_2_ surfaces. Regardless of whether or not the parylene had been completely removed, the presence of carbon on the ablated SiO_2_ surfaces would still be expected in the form of adventitious carbon [49]. The ablated SiO_2_ surfaces of parylene-N/SiO_2_ substrates contained 8.89% carbon which was consistent with the levels of 10.21%, 8.77%, and 8.36% obtained for ablated parylene-HT, parylene-D and parylene-C substrates, respectively. Similarly, the carbon 1s spectra for SiO_2_ on parylene-HT, parylene-D and parylene-C substrates, shown in Fig 3(e), 3(f) and 3(g) respectively, have similar peak shapes as the carbon 1s spectra for SiO_2_ on parylene-N substrates, shown in Fig 3(h). The similar peak shapes indicate that the carbon on ablated SiO_2_ surfaces from parylene-N substrates is likely adventitious carbon, supporting the complete removal of parylene-N.

### Effect of piranha acid treatment on parylene derivatives

**Fig 4:**
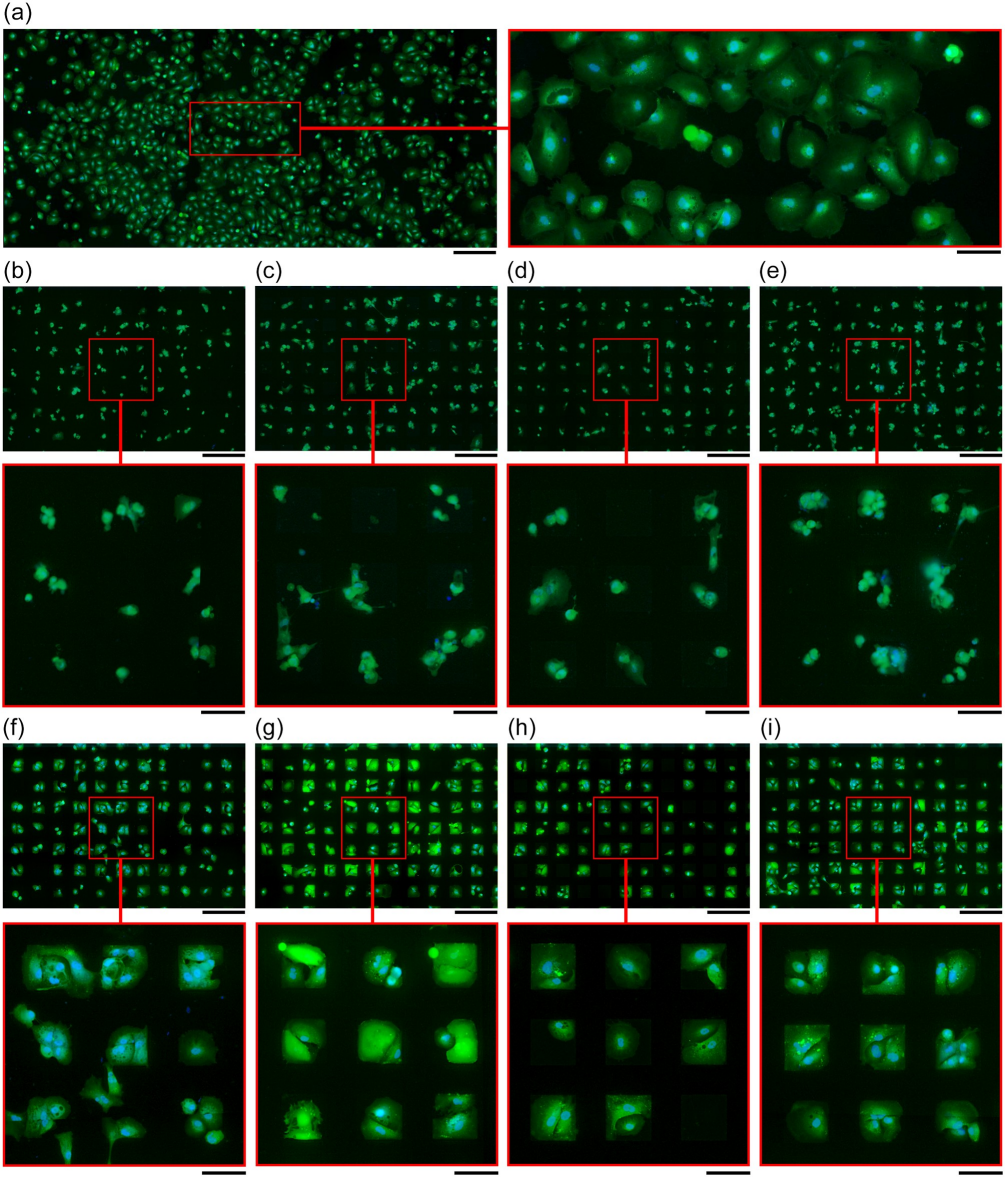
The effect of piranha acid treatment on astrocyte patterning on parylene/SiO_2_ substrates. (a) Typical morphology of hNT astrocyte in non-patterned culture. (b-e) Cell patterning on substrates prepared without the piranha acid treatment. (f-i) Cell patterning on substrates prepared with the piranha acid treatment. (b, f) Parylene-HT, (c, g) Parylene-D, (d, h) Parylene-N and (e, i) Parylene-C. Astrocyte cytoplasms were labelled with CMFDA (Green) and the nuclei with Hoechst 33258 (Blue). Overview scale-bar = 500 μm. Magnified scale-bar = 300 μm.

**Fig 5:**
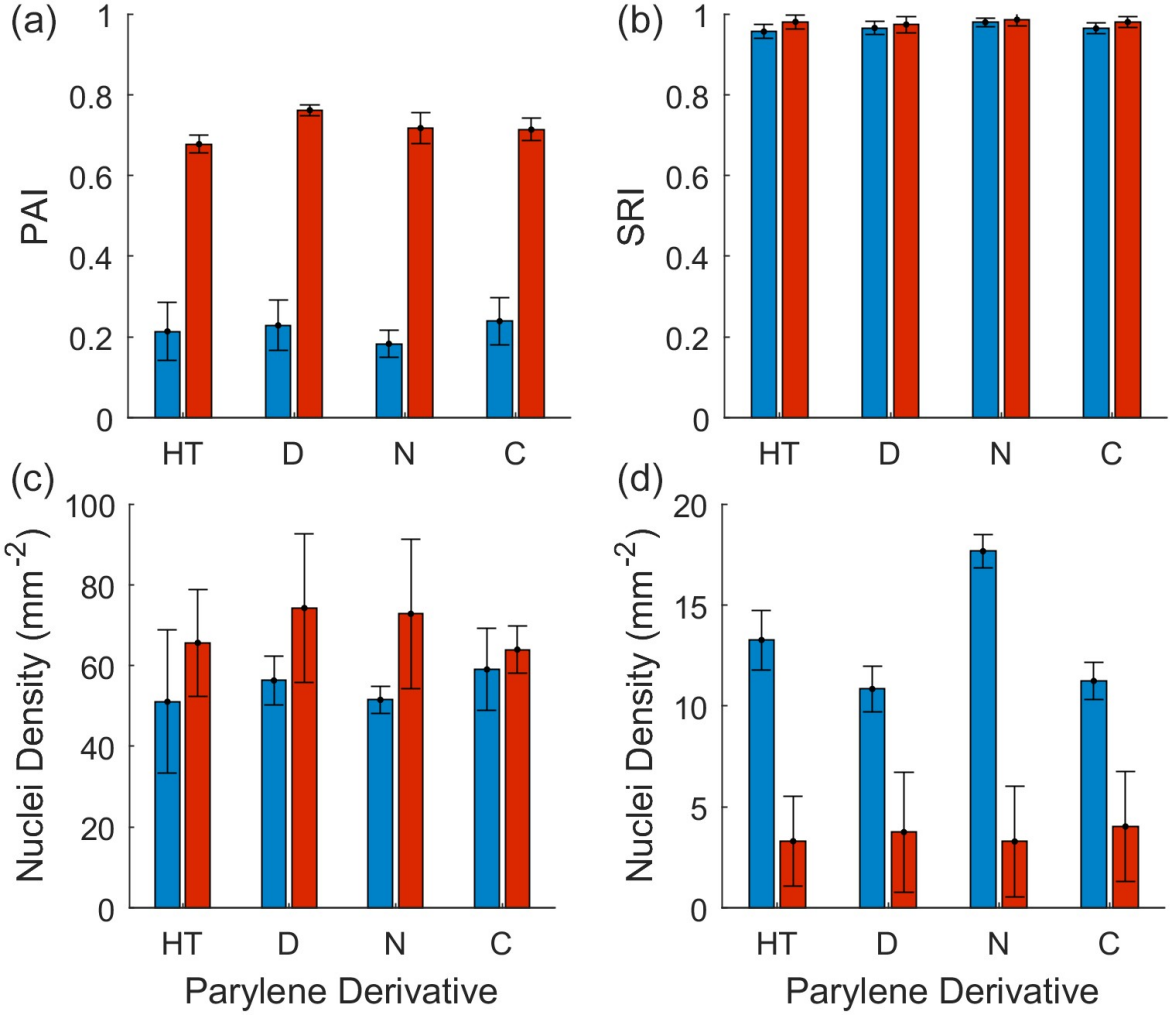
Influence of piranha acid treatment on the quality of cell patterning on parylene derivatives. Blue—No piranha acid treatment, Red—Piranha acid treated. (a) Parylene Adhesion Index. (b) Silicon Repulsion Index. (c) Nuclei density on parylene areas. (d) Nuclei density on SiO_2_ areas. Error bars represent the standard error (n = 3). Statistics and p-values are listed in Table 1.

We then evaluated the influence of piranha acid on the parylene materials. Fig 4 demonstrates, qualitatively, the influence of piranha acid treatment on serum immersed parylene substrates. The effect of the piranha acid treatment on each substrate is then quantified in Fig 5 by using the PAI and SRI metrics and considering the nuclei density on parylene and SiO_2_ areas respectively.

The typical morphology of the hNT astrocytes is characterised by a large spreading cytoplasm and is shown in Fig 4(a) [7]. Fig 4(b)–4(e) demonstrate that astrocytes cultured on parylene-HT, parylene-D, parylene-N and parylene-C, respectively, adopted an atypical morphology in the absence of the piranha acid treatment. While the astrocytes were observed to attach to the parylene surfaces, their cytoplasm did not spread across the parylene surface. In contrast, Fig 4(f)–4(i) demonstrate that astrocytes cultured on piranha acid treated parylene-HT, parylene-D, parylene-N and parylene-C, respectively, adopted a more typical morphology, although the morphology was constrained by the parylene geometry.

Fig 5 and Table 1 quantify the effect of the piranha acid treatment on astrocyte adhesion and patterning fidelity through the PAI and SRI. Fig 5(a) demonstrates that the PAI was significantly greater for every parylene derivative that was treated with piranha acid (p = 0.0036, 0.0011, 0.0004 and 0.0019 for parylene-HT, -D, -N and -C, respectively). In contrast, Fig 5(b) demonstrates that there was not any difference in the SRI for any parylene derivative regardless of whether or not the substrates were cleaned in piranha acid prior to serum immersion (p = 0.15, 0.60, 0.58 and 0.23 for parylene-HT, -D, -N and -C, respectively).

**Table 1:** P-values and null hypotheses relating to Fig 5.

*H*_0_	Fig 5(a)	Fig 5(b)	Fig 5(c)	Fig 5(d)
	*p*	*p*	*p*	*p*
*μ*_*HT w*/*o Piranha*_ = *μ*_*HT w*/ *Piranha*_	0.0036	0.15	0.55	0.021
*μ*_*D w*/*o Piranha*_ = *μ*_*D w*/ *Piranha*_	0.0011	0.60	0.41	0.0090
*μ*_*N w*/*o Piranha*_ = *μ*_*N w*/ *Piranha*_	0.0004	0.58	0.32	0.0072
*μ*_*C w*/*o Piranha*_ = *μ*_*C w*/ *Piranha*_	0.0019	0.23	0.70	0.067
*μ*_*All w*/*o Piranha*_ = *μ*_*All w*/ *Piranha*_	4.4x10^-14^	0.037	0.081	1.5x10^-6^

Next, we calculated the nuclei density on the parylene and SiO_2_ areas in order to determine whether the differences in the astrocyte adhesion could be attributed solely to differences in astrocyte morphology, rather than a difference in the number of cells. Fig 5(c) demonstrates that, for each parylene derivative, the piranha acid treatment did not result in a significant difference in the number of nuclei on parylene areas (p = 0.55, 0.41, 0.32 and 0.70 for parylene-HT, -D, -N and -C, respectively). However, we also observed that the abnormal astrocyte morphology made it difficult for the image processing algorithm to resolve individual nuclei. S1 Fig. demonstrates that, in some cases the astrocyte nuclei were too closely packed to be resolved as individual nuclei, resulting in an artefactual reduction in the nuclei density. That situation did not occur when the astrocytes grew on piranha acid treated substrates because the astrocyte cytoplasm was larger, resulting in greater separation between nuclei. However, because the differences in the nuclei density between the piranha acid treated and non-piranha acid treated substrates were not significant, we attribute the increase in the astrocyte adhesion (PAI) after piranha acid treatment to the increase in astrocyte cytoplasm spreading seen in Fig 4, rather than a difference in the nuclei density.

In contrast, Fig 5(d) demonstrates that the piranha acid treatment resulted in a significant decrease in the number of cells on SiO_2_ for piranha acid treated substrates (p = 0.021, 0.0090, 0.0072 and 0.067 for parylene-HT, -D, -N and -C, respectively). Interestingly, Fig 5(b) revealed that there was no difference in the SRI before and after piranha acid treatment, yet there is a difference in the nuclei density on SiO_2_. Similar to the effect of piranha acid on parylene there may have a greater number of astrocytes with a smaller morphology on non-piranha treated SiO_2_, whereas the piranha treatment reduced the number of astrocytes on SiO_2_ but results in greater cell spreading.

The results we present here are similar to those presented by Hughes et al. who investigated the effect of piranha acid by patterning of HEK293 cells on parylene-C/SiO_2_ substrates. Hughes et al. found that substrates that were prepared without a piranha acid treatment had lower cell attachment to the parylene-C. However, Hughes et al. also found that substrates that were prepared without piranha acid also had significant cell attachment to the SiO_2_ surfaces, resulting in a failure of the cells to pattern. In contrast, our substrates that were prepared without piranha acid did result in patterning because the SRI was not significantly different from piranha acid treated substrates. However, we also reported that the nuclei density on piranha treated SiO_2_ was significantly lower than non-piranha acid treated SiO_2_. Our results, therefore, remain consistent with those of Hughes et al. and reinforce the need for the piranha acid treatment to increase the cell repulsive characteristics of SiO_2_, increase the cell adhesive characteristics of parylene and maintain normal cell morphologies.

### Comparison of patterning quality on parylene derivatives

**Fig 6:**
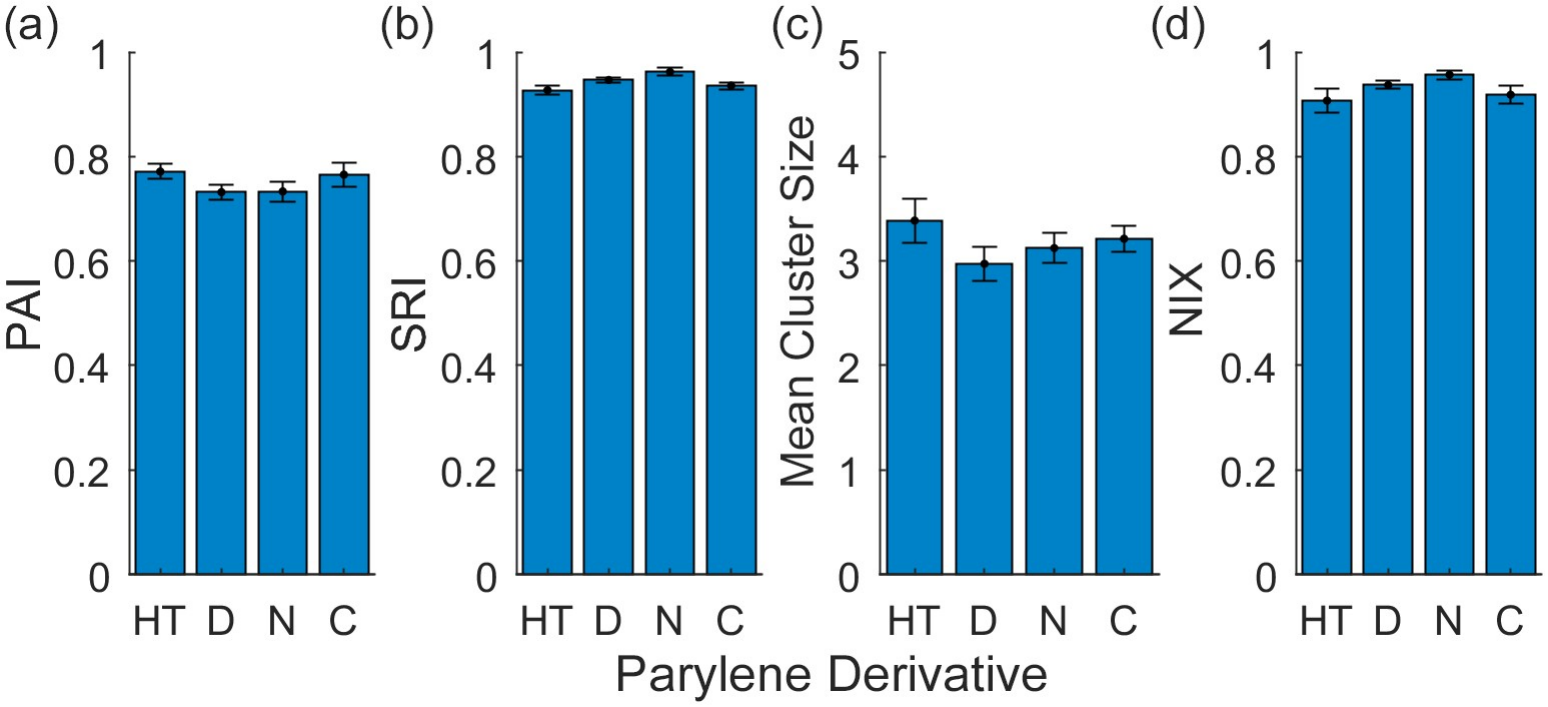
Quantification of astrocyte patterning on parylene-HT, parylene-D, parylene-N and parylene-C substrates. (a) PAI, (b) SRI, (c) Mean Cluster Size, and (d) the NIX.

We then evaluated whether there was any difference in patterning quality on parylene/SiO_2_ substrates that were made with parylene-HT, parylene-D, parylene-N or parylene-C. Fig 6 and Table 2 quantify the quality of patterning terms of cell adhesion (PAI), patterning fidelity (SRI), the mean size of the astrocyte clusters, and the isolation of astrocyte clusters (NIX).

**Table 2:** P-values and null hypotheses relating to Fig 6.

	*H*_*0*_	*p*
Fig 6(a)	*μ*_*HT*_ = *μ*_*D*_ = *μ*_*N*_ = *μ*_*C*_	0.29
Fig 6(b)	*μ*_*HT*_ = *μ*_*D*_ = *μ*_*N*_ = *μ*_*C*_	0.09
Fig 6(c)	*μ*_*HT*_ = *μ*_*D*_ = *μ*_*N*_ = *μ*_*C*_	0.36
Fig 6(d)	*μ*_*HT*_ = *μ*_*D*_ = *μ*_*N*_ = *μ*_*C*_	0.14

Fig 6(a) demonstrates that there was no significant difference in cell adhesion for any parylene derivative with all substrates achieving a PAI of approximately 0.75 (p = 0.29). Similarly, Fig 6(b) demonstrates that there was no significant difference in patterning fidelity, with all substrates achieving a SRI of approximately 0.9 (p = 0.09). We then analysed the mean cluster size and the NIX and determined that there were no significant differences between the four parylene derivatives (Fig 6(c) and 6(d)). All substrates resulted in a mean cluster size of approximately 3 and a NIX of approximately 0.9 (p = 0.36 and 0.14, respectively). These results contradict our original hypothesis that the differences in the hydrophilicity between the parylene derivatives would lead to higher fidelity patterning.

Although we did not observe an improvement in the quality of cell patterning, other authors have observed differences in cell attachment to various parylene derivatives. Kato et al. investigated the adhesiveness of PC12 cells to parylene-C and three other parylene derivatives with aromatic substitutions of amine (Parylene-A), aldehyde (Parylene-H), and alkyl amine functional groups (Parylene-AM) [42]. The parylene surfaces were used in their native state. Kato et al. observed no cell attachment to parylene-C or parylene-A, however, cell attachment was observed to parylene-AM and parylene-H. This work was expanded on by Hoshino et al. who examined the outgrowth of neurites from PC12 cells on parylene-AM and parylene-H [50]. Hoshino et al. found that neurite outgrowth was greatest on the parylene-H. Similarly, Liaqat et al. compared the proliferation and differentiation of PC12 cells on parylene-C and parylene-N surfaces that were both untreated and UV-irradiated [51]. Liaqat et al. found that untreated parylene-C supported greater cell attachment than untreated parylene-N. However, when both parylene surfaces were treated with UV light the cell adhesion increased, relative to the non-treated surfaces, and the difference in cell adhesion between parylene-C and parylene-N disappeared.

### Astrocyte calcium signalling

**Fig 7:**
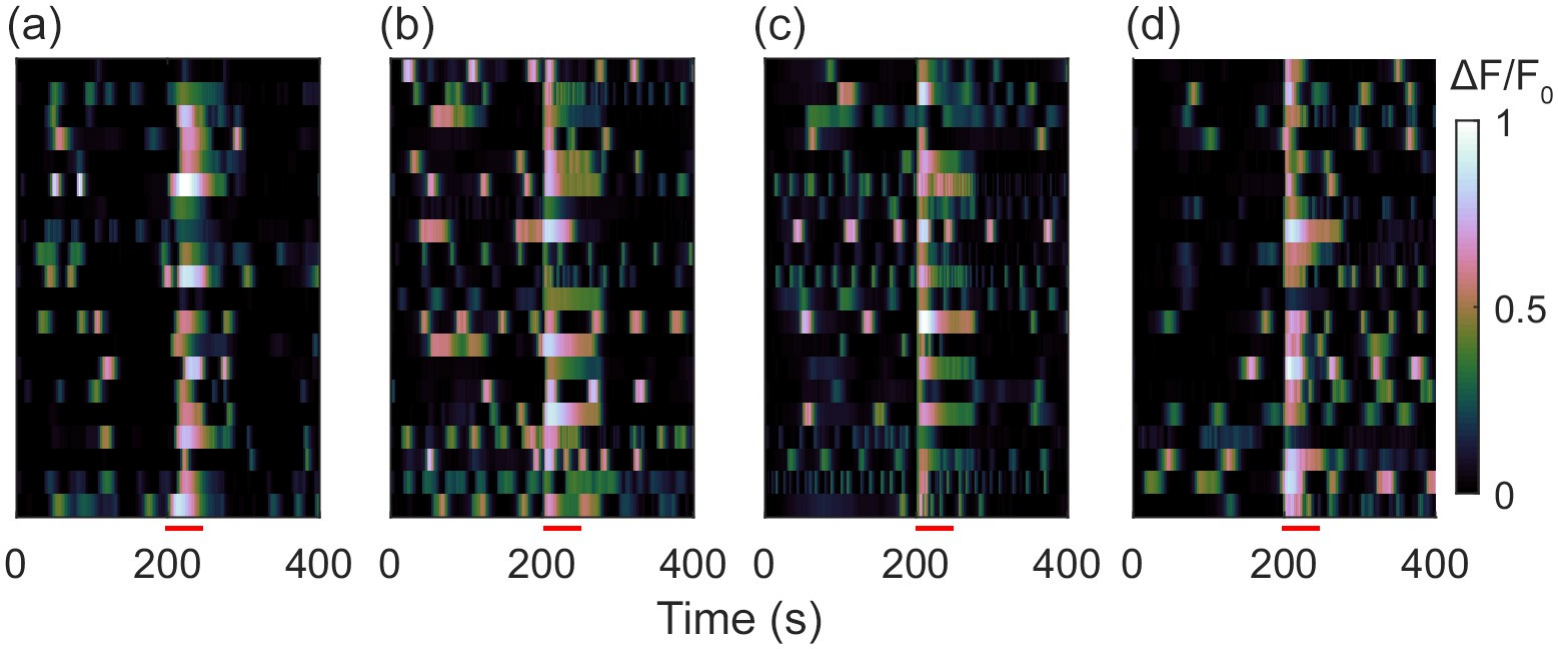
Calcium signalling in patterned hNT astrocytes. Temporal raster plots of Ca^2+^ signalling in the astrocytes patterned on (a) parylene-HT, (b) parylene-D, (c) parylene-N, (d) parylene-C/SiO_2_ substrates. Astrocytes were stimulated with 10 μm ATP at 200 s, as indicated by the red bar.

Finally, we verified that patterned astrocytes on parylene-HT, parylene-D, parylene-N and parylene-C/SiO_2_ substrates expressed functional Ca^2+^ behaviour that is normally seen in cultured astrocytes. Specifically, we verified the presence of spontaneous Ca^2+^ transients, and a robust increase in intracellular Ca^2+^ response to stimulation with ATP. Fig 7 shows temporal raster plots of the Ca^2+^ activity of 20 astrocytes patterned on parylene-HT, parylene-D, parylene-N and parylene-C/SiO_2_ substrates. Each horizontal line in the Fig 7 subplots represents the Fluo-4 fluorescence time-series from a single astrocyte. Astrocytes were imaged for 200 s, then stimulated with 10 μm ATP. We observed that the majority of astrocytes displayed spontaneous Ca^2+^ transients in the 200 s prior to ATP stimulation. Subsequently, upon application of 10 μM of ATP at 200 s virtually all astrocytes on parylene-HT, parylene-D, parylene-N and parylene-C/SiO_2_ substrates displayed an immediate increase in intracellular Ca^2+^. After the ATP induced Ca^2+^ had subsided, the astrocytes returned to their initial behaviour, exhibiting spontaneous periodic Ca^2+^ transients.

## Conclusion

In this paper, we evaluated three commercially available parylene derivatives for their use in the parylene/SiO_2_ cell patterning platform. First, we demonstrated that existing femtosecond laser manufacturing techniques are compatible with each parylene derivative. Next, we reinforced the necessity of the piranha acid treatment in both improving the contrasting cell adhesive and cell properties of parylene and SiO_2_ to enable better cell patterning, and enabling the astrocytes to adopt a more typical cell morphology. Finally, we determined that parylene-HT, parylene-D and parylene-N are all suitable for use as a cell attractive substrate and result in the same quality of patterning as parylene-C. Furthermore, all materials supported Ca^2+^ signalling that is consistent with that observed previously on parylene-C. Although we did not find a difference in the patterning of astrocytes, the processes governing cell attachment to surfaces are multifactorial and are different between different types of cells. It is therefore possible that other cells that are more sensitive to cell attachment factors may achieve higher quality patterning using a different parylene derivative on the parylene/SiO_2_ platform. It should also be noted that whilst each of the 3 parylene derivatives patterning quality was equivalent that each derivative holds different material properties which may be useful for different applications. This work provides a foundation for expanding the utility of the parylene family and the parylene/SiO_2_ patterning technology for more advanced applications.

## Supporting information

S1 Fig Abnormal astrocyte morphology on non-piranha treated substrates. (a) Fluorescence image showing astrocyte nuclei labelled with Hoechst 33258 (Blue). (b) Image processing algorithm results in a mask where not all nuclei are resolved. Scale-bar = 150 μm.
(TIF)

S1 Table Supplementary data. Raw data for XPS spectra of parylene and SiO_2_ surfaces. Data required to generate all graphs and statistical comparisons in the article.
(XLSX)
