# Supplementary figures and images for: Evaluation of parylene derivatives for use as biomaterials for human astrocyte cell patterning
Source: PLoS One. 2019 Jun 25;14(6):e0218850. doi: 10.1371/journal.pone.0218850 (PMC6592558; doi:10.1371/journal.pone.0218850)

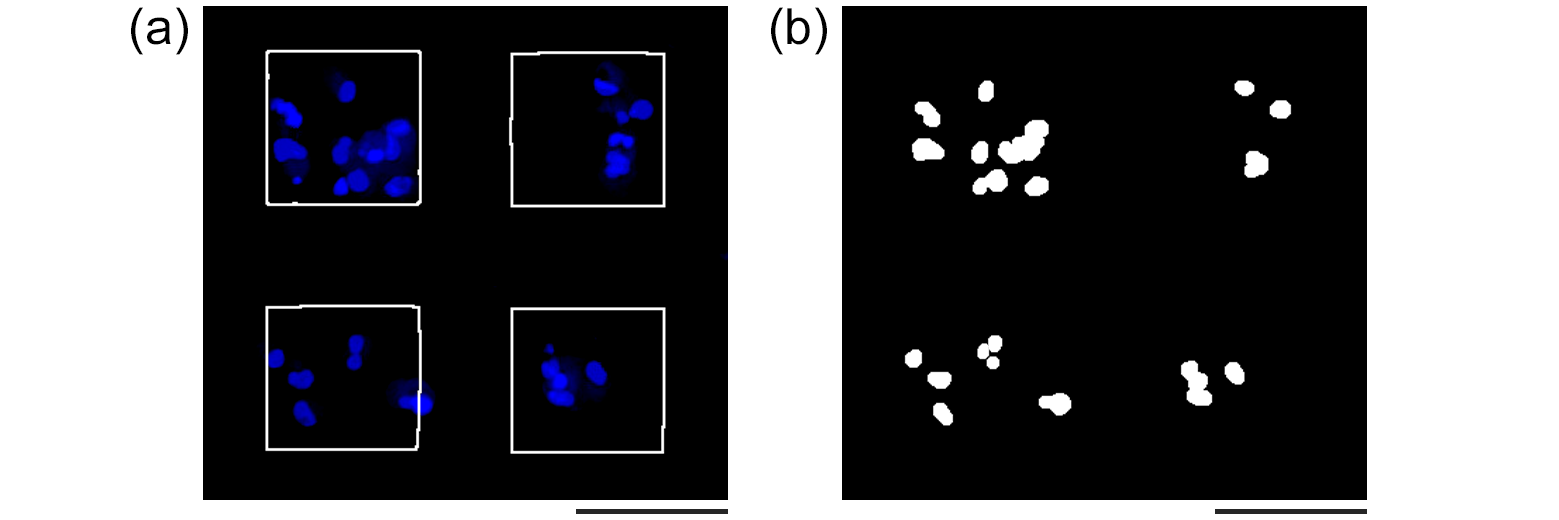

Supplement: S1 Fig — (a) Fluorescence image showing astrocyte nuclei labelled with Hoechst 33258 (Blue). (b) Image processing algorithm results in a mask where not all nuclei are resolved. Scale-bar = 150 μm. (TIF) [file pone.0218850.s001.tif]
